# Joint Covariate Detection on Expression Profiles for Selecting Prognostic miRNAs in Glioblastoma

**DOI:** 10.1155/2017/3017948

**Published:** 2017-03-20

**Authors:** Chengqi Sun, Xudong Zhao

**Affiliations:** College of Information and Computer Engineering, Northeast Forestry University, Harbin 150001, China

## Abstract

An important application of expression profiles is to stratify patients into high-risk and low-risk groups using limited but key covariates associated with survival outcomes. Prior to that, variables considered to be associated with survival outcomes are selected. A combination of single variables, each of which is significantly related to survival outcomes, is always regarded to be candidates for posterior patient stratification. Instead of individually significant variables, a combination that contains not only significant but also insignificant variables is supposed to be concentrated on. By means of bottom-up enumeration on each pair of variables, we propose a joint covariate detection strategy to select candidates that not only correspond to close association with survival outcomes but also help to make a clear stratification of patients. Experimental results on a publicly available dataset of glioblastoma multiforme indicate that the selected pair composed of an individually significant and an insignificant miRNA keeps a better performance than the combination of significant single variables. The selected miRNA pair is ultimately regarded to be associated with the prognosis of glioblastoma multiforme by further pathway analysis.

## 1. Introduction

Survival analysis, which is a branch of statistics for analyzing time-to-event data, is commonly used in cancer research. In particular, it helps to assess the prognosis of patients having specific types of cancer in informing not only the categories of patients with differing survival outcomes but also the possible molecular cause of the risk of death. Narrow down to gliomas, expression profiles are utilized to discover the subtypes of patients with different survival risks [1]. This kind of data provides a supplementary predictor of survival due to the limited effectiveness of current clinical diagnoses. Numerous studies which attempted to use selected signatures from expression profiles for discrete stratification (e.g., recurrence, metastasis, and chemotherapy efficacy) have shown the effectiveness. Correspondingly, several methods that classified patients into subgroups with differing survival time have also been performed.

Considering the continuity of the observations' survival time with right censoring, Cox proportional hazards regression analysis [2] was extensively utilized to seek covariates associated with the overall survival of patients in invasive breast cancer [3], non-small-cell lung cancer [4], follicular lymphoma [5], glioblastoma [6–8], and so forth. Due to the requirement of more observations than covariates, Cox proportional hazards regression model was combined with some methods for dimension reduction or shrinkage such as partial least squares [9] and principle component analysis [10]. However, these strategies can only provide a combination of variables other than reporting meaningful covariates. Since projections derived from these variables are made, one can only tell these variables together but not which variables are effective. Besides, top-down methods of tree-structured survival analysis [11] and random survival forests [12] associated with hazards regression were proposed for selection of covariates. Unlike bottom-up enumeration strategies, these heuristic approaches may get local optimal solutions although they infinitely approximate to global optimal solutions despite their efficiencies.

Hence, univariable regression analyses have been placed firmly in the mainstream. Due to the high-dimensional space of variables compared to the small observation size, a penalized Cox hazards model using least-angle regression was proposed in order to solve the overfitting problem of parameter learning [13]. In addition, a sparse kernel method was proposed on condition that the correlation between the logarithm of the hazard ratio and covariates was linear, and a survival supporting vector machine that maximized the classification margin other than Cox regression was presented [14]. In practice, univariable Cox regression analysis was applied to each variable, which was regarded to be significant considering its correlation with survival time or its distinct stratification of patients. Significant variables were selected using either Wald *t*-test on regression coefficients [15] or log-rank test with permutations after dividing patients into high- and low-risk groups by univariable risk-score analysis [16, 17]. A risk score of each observation was obtained using a linear combination of the expression levels of selected variables weighted by multivariable regression coefficients. A cut-off threshold was derived from the median risk score or was determined by receiver operating characteristics (ROC) analysis [18], and patients within the training set were divided into high- and low-risk groups. The major problem of univariable Cox regression strategy roots in the assumption that covariates are derived from individual variables, each of which is significantly associated with survival outcomes. In essence, a meaningful set of covariates are probably composed of different variables, each of which is either correlated with or apparently unrelated to survival outcomes.

In order to solve this problem, we propose a joint covariate detection strategy for selection of variable pairs instead of a combination of variables individually correlated with survival time from expression profiles. Meanwhile, stratification of patients is also considered. That is, predictors not only associated with survival outcomes but also helpful to classify patients into high-risk and low-risk groups are chosen. Cox proportional hazards regression is used in order to detect variable pairs that are most associated with survival time. In order to overcome the overfitting problem, variable pairs which may most possibly help to stratify patients with differing survival risks are further selected. In particular, patients are stratified according to the corresponding risk-score analysis derived from Cox proportional hazards regression. Besides, log-rank test is performed for further confirmation whether the selected variable pairs contribute mainly to the stratification or not. In order to show the effectiveness of our method, miRNA expression profiles containing 548 patients with glioblastoma multiforme (GBM) downloaded from the Cancer Genome Atlas (TCGA) database are introduced in. The final selected miRNA pair of significance as representing the covariates not only most associated with survival outcomes but also effective to stratification of patients is ultimately testified using KEGG pathway analysis.

## 2. Materials and Methods

### 2.1. Microarray Data

We use the miRNA expression data (Level 3) of 548 patients with GBM downloaded from TCGA (http://cancergenome.nih.gov) in order to illustrate the effectiveness of identifying prognostic miRNAs in glioblastoma using the joint covariate selection method. In total, these 548 GBM cases with overall survival information are selected from 581 miRNA expression profiles, which were downloaded during May, 2015. We choose all the patients, for we discover that splitting samples using a random dichotomy or by balancing survival outcomes between training and testing group cannot achieve the same set of variables as it is derived from the whole samples. That is to say, how to reasonably split samples into training and testing ones is still under discussion. The reason derives from two aspects. One is that survival outcomes are continuous compared to discrete stratification (e.g., recurrence, metastasis, and chemotherapy efficacy). Thus, the distribution of survival outcomes is to be estimated before splitting samples. The other is that it is hard to estimate the distribution of survival outcomes because of including censored following time. Moreover, the survival time of each patient is recorded, which ranges between 0 and 3881 days. Among them, 450 are dead (uncensored) during the study and 98 are still alive (censored) at the end of the study.* MatlabR2013b* is selected as the experimental platform. Coefficients of Cox regression are obtained by calling the library function* coxphfit*.

### 2.2. Joint Covariate Detection

Here, it represents a twofold consideration on detection of variables, which are both associated with survival outcomes and helpful to classify patients into different risk groups. In order to seek variables associated with survival outcomes, Cox hazards regression is firstly introduced. The partial likelihood function is given by the expression

(1) $$\begin{array}{c} l\left(\;\beta \;\right)=\overset{m}{\underset{i=1}{\prod }}\frac{e^{x_{\left(i\right)}^{T}\beta }}{\sum_{j\in R\left(t_{\left(i\right)}\right)}e^{x_{j}^{T}\beta }}, \end{array}$$

where the product is over the *m* distinct ordered survival time without any follow-up of right censoring assuming that there is no tied time. **x**_(*i*)_ and **β** denote the *i*th expression levels and the regression coefficients of the detected variables, respectively. The summation in the denominator is over all subjects in the risk set at ordered survival time *t*_(*i*)_, denoted by *R*(*t*_(*i*)_). The maximum partial likelihood estimator is obtained by differentiating the right hand side of the logarithm transformation of (1) with respect to **β**, setting the derivative equal to zero, and solving for **β**. As to each component of **β**, a Wald statistic that represents the ratio of the estimated coefficient to its estimated standard error is presented. That is,

(2) $$\begin{array}{c} z_{k}=\frac{{\hat{\beta }}_{k}}{\hat{SE}\left({\hat{\beta }}_{k}\right)}. \end{array}$$

The *p* value of the *k*th component of **β** is obtained by looking up a table assuming that the Wald statistic in (2) follows the standard normal distribution. In order to enlarge the sample size, we make a permutation test by reordering the survival outcomes for *B* times. And the corresponding *p* value is expressed as follows:

(3) $$\begin{array}{c} p_{k}=\overset{B}{\underset{b=1}{\sum }}\frac{\#\left(\left|z_{k}^{0}\right|\geq \left|z_{k}\right|\right)}{B}, \end{array}$$

where *z*_*k*_^0^ denotes a null statistics by a random rearrangement of survival outcomes. Enumeration on each single variable or on each pair is made. Therefore, covariates significantly associated with survival outcomes are selected according to the individuals or the pairs with smallest *p* values.

Meanwhile, we consider a best stratification of patients with differing survival outcomes as an indicator for selection of covariates. In practice, patients are commonly classified into low-risk and high-risk groups, which conforms to the daily doctors' decision making process. Following the case, the risk score is the linear portion of Cox regression model, of which the estimator for the *i*th sample containing *p* covariates is

(4) $$\begin{array}{c} {\hat{r}}_{i}=\hat{r}\left(\;x_{i},\hat{\beta }\;\right)=\overset{p}{\underset{k=1}{\sum }}{\hat{\beta }}_{k}x_{ik}. \end{array}$$

Median risk score is utilized as a cut-off value for stratification, in order to keep the equivalent number between high-risk and low-risk patients. Assuming that the survival function is the same in each of the two groups, the estimator of the expected number of deaths in high-risk group is expressed as

(5) $$\begin{array}{c} {\hat{e}}_{1i}=\frac{n_{1i}d_{i}}{n_{i}}, \end{array}$$

where *n*_*i*_ and *d*_*i*_ represent the number at risk and of deaths at the observation of ordered survival time *t*_(*i*)_, respectively. *n*_1*i*_ denotes the number at risk in high-risk group. Correspondingly, the estimator of the variance of *d*_1*i*_ on the hypergeometric distribution is defined as follows:

(6) $$\begin{array}{c} {\hat{v}}_{1i}=\frac{n_{1i}n_{0i}d_{i}\left(n_{i}-d_{i}\right)}{n_{i}^{2}\left(n_{i}-1\right)}, \end{array}$$

where *n*_0*i*_ denotes the number at risk in low-risk group. Under the null hypothesis that survival functions of the two groups are the same, the statistic of log-rank test is expressed as follows:

(7) $$\begin{array}{c} Q=\frac{{\left[\sum_{i=1}^{m}\left(d_{1i}-{\hat{e}}_{1i}\right)\right]}^{2}}{\sum_{i=1}^{m}{\hat{v}}_{1i}}. \end{array}$$

The corresponding *p* value is obtained using the *χ*^2^ distribution with one degree of freedom. In the same way, we make a permutation test similarly expressed in (3). That is,

(8) $$\begin{array}{c} p_{r}=\overset{B}{\underset{b=1}{\sum }}\frac{\#\left(\left|Q_{r}^{0}\right|\geq \left|Q_{r}\right|\right)}{B}, \end{array}$$

where *Q*_*r*_^0^ also represents a null statistics by a random rearrangement of survival outcomes. After enumerating on each individual variable or on each pair, covariates that significantly categorize patients with differing survival outcomes are detected according to smallest *p* values.

By enumeration on each variable and each pair, significant covariates most associated with survival time are chosen on condition that each component keeps a small *p* value as expressed in (3). Moreover, the variables for stratification of patients using the risk score defined by (4) correspond to small *p* values as seen in (8). In fact, this conception derives from Integrative Hypothesis Testing (IHT) proposed by Xu [19]. The obtained covariates may indicate not only a close correlation with survival time but also distinct stratification of patients.

### 2.3. KEGG Pathway Analysis

In order to show the effectiveness of our method, we submit the final selected miRNA pair, which is not only most associated with survival outcomes but also effective to stratification of patients to low-risk and high-risk groups, to DIANA miRPath [20]. We only use TarBase [21] to select the targets of the miRNA pair, considering that it is a database of published experiments validated miRNA-gene interactions. Focusing on the pathways related to the selected miRNA pair instead of those corresponding to each component of the selected pair, we can find significant pathways, which may support our finding and show the effectiveness of our method.

## 3. Results

### 3.1. Joint Covariate Detection for GBM Survival Analysis

In this part, we apply joint covariate detection to seeking miRNAs which are associated with the risk of death and the stratification of high-risk and low-risk patients in GBM. The representation of “joint” is twofold. First, it is a strategy that combines Cox regression for seeking survival-associated variables with log-rank test on risk scores for evaluation of the classification results. Second, it also exhibits the steps from enumerations on each individual variable to those on enumerable covariate tuples. Considering the computational cost, joint covariate detection terminates after finishing enumeration on miRNA pairs.

**Figure 1:**
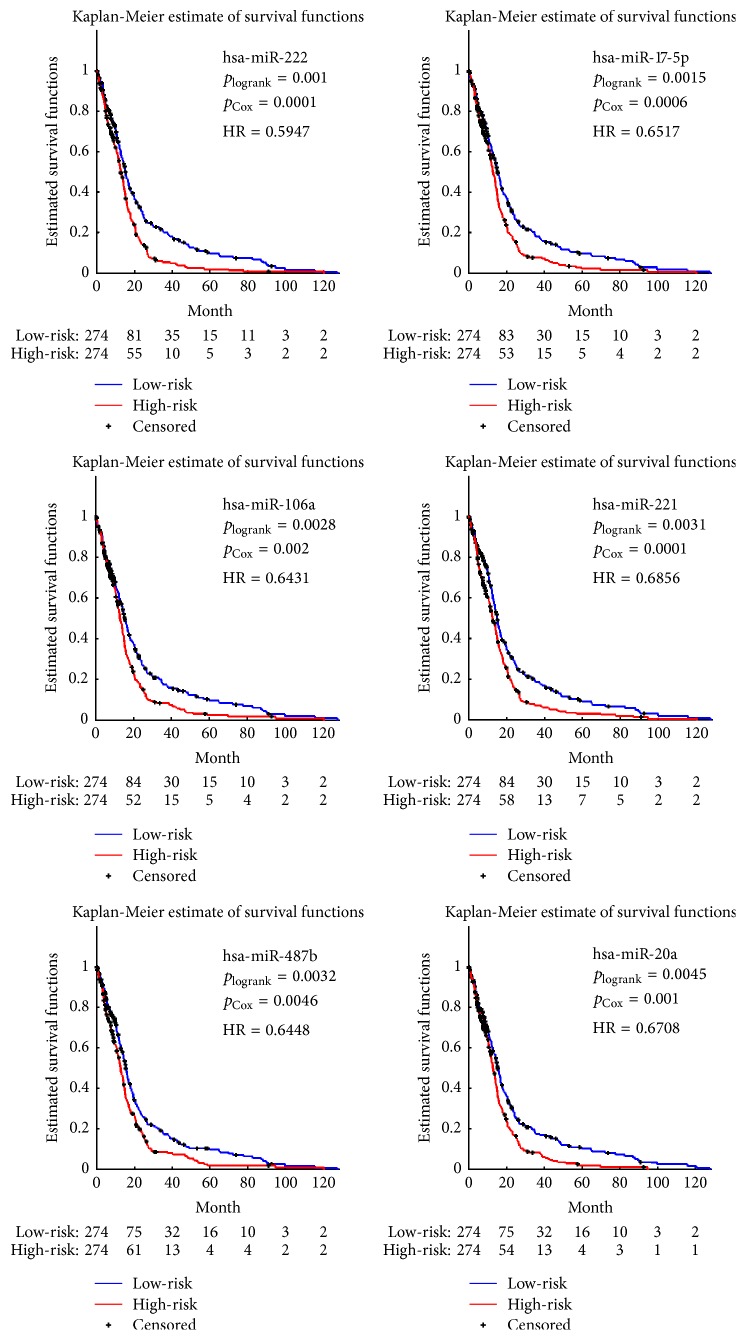
Kaplan-Meier survival analysis using significant individual miRNAs, each of which is jointly selected to Cox regression and log-rank test with *p* values ≤ 0.01.

For each miRNA, *p* values expressed in (3) and (8) were obtained after 10000 rounds of permutations. The miRNAs with *p* values ≤ 0.01 were regarded to be individually significant. We obtained six significant miRNAs, as listed in Table 1. Using the expression levels of the selected significant miRNAs, we made Kaplan-Meier survival analyses on high-risk and low-risk groups derived from cut-off values by calculating the median risk scores expressed in (4), as illustrated in Figure 1. Besides, *p* values of each significant miRNA were also shown in Figure 1. On assumption that hazard ratio (HR) is constant over survival time, we listed HRs in Figure 1, too.

**Figure 2:**
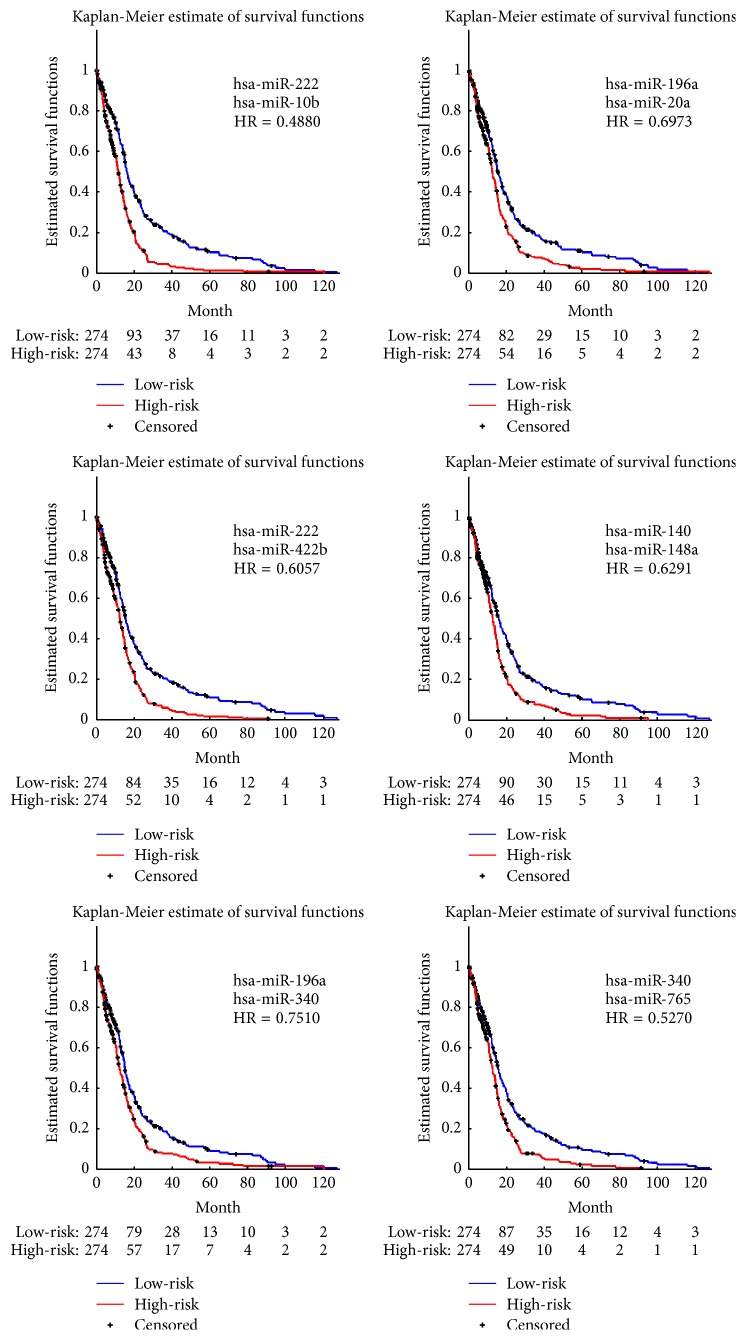
Kaplan-Meier survival analysis using significant miRNA pairs, each of which is jointly selected to Cox regression and log-rank test with *p* values ≤ 0.001.

As to each miRNA pair, permutations with 10000 rounds were made. *p* values corresponding to each component of every pair were calculated by (3). After a linear combination of the expression levels which regarded the learned Cox regression coefficients as its weights, risk scores were obtained using (4). The median risk score was utilized as a cut-off value; therefore, patients were classified into the high-risk and low-risk groups. Log-rank test expressed in (7) was performed, and the corresponding *p* value representing the significant differences of risks between the two groups was calculated by (8). The miRNA pairs with *p* values ≤ 0.001 were regarded as the significant pairs associated with the risk of death to patients in GBM. We obtained six significant pairs of miRNAs (see Table 2), of which survival analyses were shown in Figure 2.

**Figure 3:**
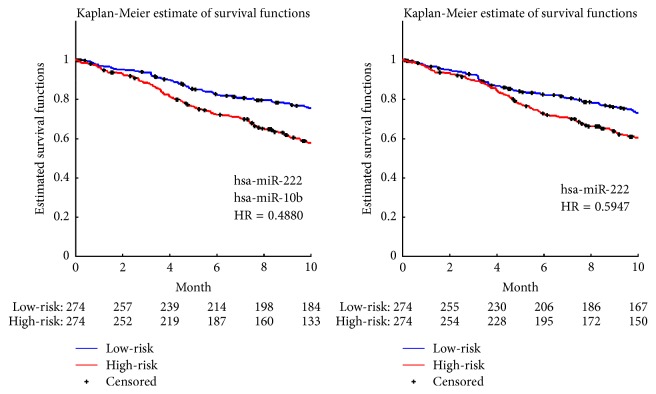
Comparisons between the most significant pair and the most significant individual miRNA.

Figure 2 illustrated the experimental results of the significant pairs. Kaplan-Meier survival analysis was made between the high-risk and low-risk groups of patients on each significant pair. *p* values of each component and that corresponding to log-rank test were also listed in Table 2. By making a careful comparison between the Kaplan-Meier curves associated with miRNA pairs shown in Figure 3 and those related to individually significant miRNAs illustrated in Figure 1, we discovered that the selected miRNA pairs contributed to an easier stratification of patients with survival months less than 10 months, as illustrated in Figure 3.

**Figure 4:**
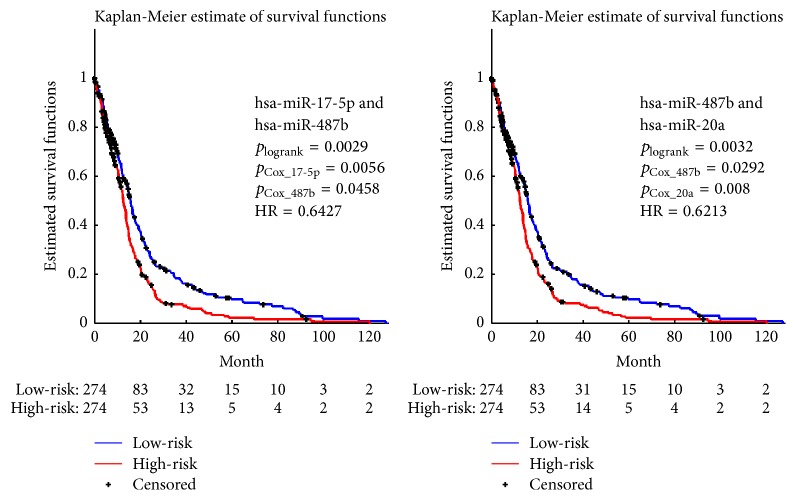
Representative survival analysis using combinations of the six significant individual miRNAs, each of which is jointly selected according to Cox regression and log-rank test with *p* values ≤ 0.05.

Next, we tried to validate that covariates most associated with survival outcomes were not equal to the set of individually significant variables. In order to demonstrate it, we enumerated all possible combinations of the six miRNAs that were individually significant as listed in Table 1 and illustrated in Figure 1 and performed joint covariate detection on each combination. We made 10000 rounds of permutations and set the threshold to be 0.05 for significant detection. Of all the 57 combinations except six individually significant miRNAs, two significant combinations were obtained, as illustrated in Figure 4. *p* values of each miRNA and that corresponding to log-rank test were listed for each combination. After carefully comparing parameters in Figure 2 with those in Figure 4, we made a conclusion that covariates selected for discrimination of GBM prognosis could not only consist of individually significant variables. In other words, significant covariates possibly consisted of different variables, each of which is either individually significant or not.

### 3.2. Verification of miRNAs Associated with GBM Prognosis

According to the small *p* values and small HRs illustrated in Figure 2, we selected the most significant miRNA pair (i.e., miR-10b and miR-222). In order to validate the chosen pair's close association with prognosis of GBM instead of significant or insignificant individuals, we used DIANA miRPath [20] and TarBase [21] which provide miRNA/gene interactions with high quality experimental validations to identify KEGG pathways related to both miR-222 and miR-10b. Pathways corresponding to miR-222, miR-10b and their combination are listed in Tables 3, 4, and 5, respectively.

**Figure 5:**
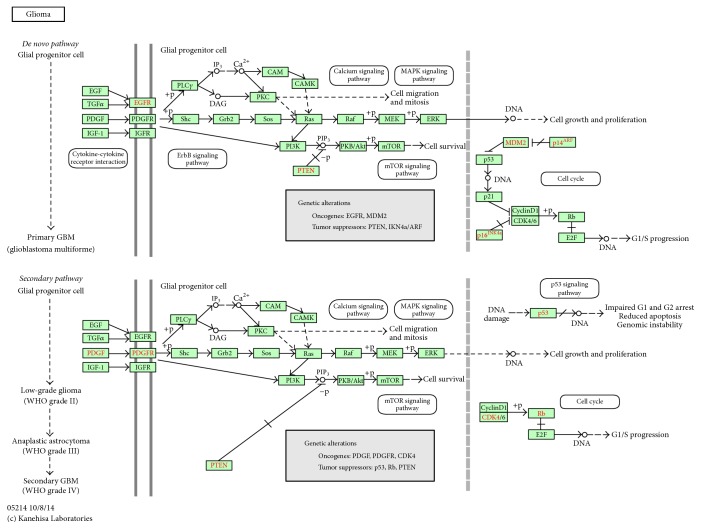
Glioma pathway that shows close association with miR-222 and miR-10b.

Comparisons from Tables 3–5 show that pathways including glioma and melanoma may have direct relations with both miR-222 and miR-10b, which might indirectly support our option about the need of joint covariate detection. The glioma pathway is illustrated in Figure 5.

## 4. Discussion

In this paper, a joint covariate detection strategy is proposed for selecting candidates that not only correspond to close association with survival outcomes but also help to make a clear stratification of patients. We choose GBM data and testify the effectiveness of our method on it for three reasons presented as follows. First, GBM data has a large sample size containing 548 patients. Such a large sample size ensures the reliability of statistical results, and that is also the reason why we keep the whole samples for training the model. Second, GBM data has a very long follow-up time, the longest of which has reached a length of over ten years. Third, right censored observations are keeping in a smaller sample size, which now has 98 cases compared to 450 having passed away. Less censored samples make more robust fitting result of Cox proportional hazards regression.

Joint covariate detection contains the concept that makes a combination between selecting covariates most associated with survival outcomes and seeking covariates which is capable of risk stratification. To the best of our knowledge, it is the first model using bottom-up enumerations of variable pairs other than combination of individually significant variables, which has been widely provided in practice. Considering that the expression profiles commonly have large dimension and small sample size, permutation tests are made by reordering survival outcomes to enlarge sample size. Besides, log-rank tests may also help to solve the overfitting problem. Model development such as covariate interaction [22] can be further introduced in.

However, several limitations of the proposed strategy have to be listed as follows. First, joint covariate detection terminates after finishing the pair enumeration due to the high computational cost. In order to proof our inference, enumerations on multituples of variables need to be done. Second, strategies which contain penalties or constraints are excluded considering a fast performing demand. Third, we keep Cox proportional hazards assumption that the hazard ratio is independent of survival time. In fact, it is the covariate but not the regression coefficient that does not depend on survival time. Covariates whose values are fixed at the beginning of observation remain unchanged throughout the follow-up time. On condition that the difference in log hazards depended on time, a nonparametric concordance measure [23] or an alternative selection of concordance regression and weighted Cox regression [24] were presented, respectively, instead of Cox regression. Once the problem of computational cost is solved, these improvements can be added in. Fourth and most important, how to reasonably split samples with survival outcomes into training and testing ones is still a vital problem under discussion. Actually, splitting samples using a random dichotomy or by balancing survival outcomes between training and testing group will not work, especially on low-dimension feature space. And that has been experimentally demonstrated (not shown). All these limitations are to be settled in the future.

Using joint covariate detection, we chose one miRNA pair associated with GBM prognosis. In order to reveal the relationship between the chosen miRNA pair and the survival time of GBM patients, DIANA miRPath [20] and TarBase [21], which provided miRNA/gene interactions with high quality experimental validations, were utilized. As listed in Table 5, two pathways including glioma and melanoma were manifested, which indicated a joint action of the miRNA pair. With experimental validations, four common targets of each component in the miRNA pair (i.e., MDM2, TP53, CDK6, and E2F3) were focused on. In Figure 5, the four targets were included in cell cycle pathway, which was also the case in melanoma pathway. MDM2 and TP53 were reported to be directly associated with prognosis of GBM [25]. As illustrated in Figure 5, INK4a and ARF acted as tumor suppressors, which were upstream genes of the common targets. The loss of INK4a and ARF together with p53 gene mutation was reported to be mutually exclusive events in GBM [26].

## Figures and Tables

**Table 1:** Individual results using joint covariate detection.

miRNA probe	*Z* (log-rank)	*p* (log-rank)	Coef (Cox)	*Z* (Cox)	*p* (Cox)	Expressions in high-risk group
hsa-miR-222	3.042262	0.0012	0.245557	6.33205	0.0001	High
hsa-miR-17-5p	2.878053	0.0015	−0.22319	−3.36403	0.0006	Low
hsa-miR-106a	2.841924	0.0028	−0.18956	−3.00119	0.002	Low
hsa-miR-221	2.792194	0.0031	0.283759	5.395549	0.0001	High
hsa-miR-487b	2.711448	0.0032	0.207506	2.849673	0.0046	High
hsa-miR-20a	2.688864	0.0045	−0.1768	−3.16297	0.001	Low

**Table 2:** Pair results using joint covariate detection.

miRNA probe	miRNA probe	*p* (log-rank)	*p* (Cox)	*p* (Cox)	Expressions in high-risk group	Expressions in high-risk group
hsa-miR-10b	hsa-miR-222	0.0002	0.0004	0.0001	High	High
hsa-miR-196a	hsa-miR-20a	0.0003	0.0007	0.0002	High	Low
hsa-miR-222	hsa-miR-422b	0.0003	0.0001	0.0007	High	Low
hsa-miR-140	hsa-miR-148a	0.0007	0.0004	0.0001	Low	High
hsa-miR-196a	hsa-miR-340	0.0007	0.001	0.0003	High	Low
hsa-miR-340	hsa-miR-765	0.0009	0.0001	0.0006	Low	Low

**Table 3:** Pathways associated with miR-222 (*p* ≤ 0.01).

KEGG pathway	*p* value	# genes	# miRNAs
Fatty acid biosynthesis	1.64*E* − 25	1	1
Fatty acid metabolism	5.15*E* − 05	2	1
Arrhythmogenic right ventricular cardiomyopathy (ARVC)	5.15*E* − 05	8	1
Viral carcinogenesis	0.000376	22	1
Protein processing in endoplasmic reticulum	0.006288	20	1
Lysine degradation	0.011993	5	1
RNA degradation	0.011993	11	1
p53 signaling pathway	0.011993	11	1
Ubiquitin mediated proteolysis	0.013637	17	1
RNA transport	0.02166	20	1
Cell cycle	0.02166	15	1
Spliceosome	0.024132	11	1
Endometrial cancer	0.038151	6	1
Adherens junction	0.041217	9	1
HTLV-I infection	0.046109	24	1
Central carbon metabolism in cancer	0.047174	6	1
Bacterial invasion of epithelial cells	0.049002	9	1

**Table 4:** Pathways associated with miR-10b (*p* ≤ 0.01).

KEGG pathway	*p* value	# genes	# miRNAs
Fatty acid biosynthesis	4.92*E* − 28	1	1
Viral carcinogenesis	1.11*E* − 06	16	1
Fatty acid metabolism	2.78*E* − 06	1	1
Chronic myeloid leukemia	0.0005	9	1
Central carbon metabolism in cancer	0.002282	7	1
Non-small-cell lung cancer	0.007224	8	1
Glycosphingolipid biosynthesis, lacto- and neolactoseries	0.015745	2	1
Pyrimidine metabolism	0.021438	7	1
Cell cycle	0.022075	13	1
p53 signaling pathway	0.028173	9	1

**Table 5:** Pathways associated with both miR-10b and miR-222 (*p* ≤ 0.01).

KEGG pathway	*p* value	# genes	# miRNAs
Fatty acid biosynthesis	5.98*E* − 47	1	2
Fatty acid metabolism	6.60*E* − 22	1	2
Viral carcinogenesis	1.54*E* − 05	5	2
Chronic myeloid leukemia	0.001129	4	2
*Glioma*	0.015536	4	2
Non-small-cell lung cancer	0.023173	4	2
*Melanoma*	0.030385	4	2
Cell cycle	0.043136	6	2
